# Draft Genome Sequence of the Deinococcus-Thermus Bacterium Meiothermus ruber Strain A

**DOI:** 10.1128/genomeA.00202-15

**Published:** 2015-03-26

**Authors:** Vera Thiel, Lynn P. Tomsho, Richard Burhans, Scott E. Gay, Stephan C. Schuster, David M. Ward, Donald A. Bryant

**Affiliations:** a Department of Biochemistry and Molecular Biology, The Pennsylvania State University, University Park, Pennsylvania, USA; b Singapore Centre for Environmental Life Sciences Engineering, Nanyang Technological University, Singapore; c Department of Chemistry and Biochemistry, Montana State University, Bozeman, Montana, USA; d Department of Land Resources and Environmental Sciences, Montana State University, Bozeman, Montana, USA

## Abstract

The draft genome sequence of the Deinococcus-Thermus group bacterium Meiothermus ruber strain A, isolated from a cyanobacterial enrichment culture obtained from Octopus Spring (Yellowstone National Park, WY), comprises 2,968,099 bp in 170 contigs. It is predicted to contain 2,895 protein-coding genes, 44 tRNA-coding genes, and 2 rRNA operons.

## GENOME ANNOUNCEMENT

Meiothermus ruber strain A is a moderately thermophilic, aerobic, and heterotrophic bacterium of the family *Thermaceae* of the phylum Deinococcus-*Thermus*. Strain A was isolated from an enrichment culture of Synechococcus sp. strain JA-3-3-Ab, which was cultivated from a phototrophic microbial mat in an effluent channel of Octopus Spring, an alkaline siliceous hot spring in Yellowstone National Park (44°32′2.833″N 110°47′53.352″W, WY, USA) (1, 2). The enrichment culture grew in liquid BG11 medium (3), while BG11 supplemented with 0.01% (wt/vol) yeast extract and solidified with 0.2% Phytagel (Sigma-Aldrich) was used to isolate strain A. The 16S rRNA sequence of this isolate shares 98.6% nucleotide identity to the 16S rRNA gene sequence of M. ruber DSM 1279^T^.

Purified genomic DNA from M. ruber strain A was sequenced using an Illumina MiSeq instrument and assembled with Newbler (version 2.9; Roche) from 3,082,447 301-bp paired reads. The assembly used for genome analysis contained 170 contigs with a minimum length of 500 bp, comprising 2,968,099 bp, with an average G+C content of 63% and a median coverage of 476-fold.

Annotation using RAST (4) predicted 2,895 protein-coding genes, 44 tRNA-coding genes, and 2 rRNA operons, compared to 3,052 protein coding genes, 53 RNAs, and 2 rRNA operons found in the M. ruber type strain DSM1279^T^ genome (5). Phyla-AMPHORA (6) and manual BLASTp searches identified 508 of 517 Deinococcus-*Thermus*-specific phylogenetic marker genes. This suggests that the draft genome is >90% complete and that the genome is comparable in size to that of other M. ruber genomes (3.03 to 3.10 Mb [5, 7]).

Consistent with the growth characteristics of this isolate, as well as those of the type strain, genes encoding all enzymes associated with glycolysis, the tricarboxylic acid (TCA) cycle, the Entner-Doudoroff and pentose phosphate pathways, as well as a complete respiratory electron transport chain, including NADH dehydrogenase, succinate dehydrogenase, ubiquinol:cytochrome *c* oxidoreductase (cytochrome *bc*_1_), and two cytochrome *c* oxidases (*aa*_3_ and *ba*_3_-type) are present. These findings are consistent with the identification of this organism as an aerobic heterotroph (8). The genome lacks genes for nitrate reduction and nitrogen fixation, which suggests that this strain depends on reduced nitrogen sources (8).

Similar to other M. ruber isolates, strain A is pink in color, due to the presence of carotenoids. The major pigment in one strain of M. ruber was previously identified as 1′-β-glucopyranosyl-3,4,3′,4′-tetradehydro-1′,2′-dihydro-β,ψ-caroten-2-one (9). The genes *crtBI* and *crtY* for carotenoid biosynthesis are present in the partial genome, suggesting the capability to produce the backbone β,ψ-carotene, while a putative *crtO*-type β-carotene ketolase is probably responsible for the introduction of the 2-one moiety. Together with high-performance liquid chromatography data, the presence of two additional phytoene dehydrogenases, as well as a gene encoding a glycosyltransferase adjacent to a phytoene dehydrogenase and a putative acyltransferase gene, supports the hypothesis that strain A produces carotenoid glycoside esters similar to those previously identified (9).

### Nucleotide sequence accession numbers.

This whole-genome shotgun project has been deposited at DDBJ/EMBL/GenBank under the accession no. JXOP00000000. The version described in this paper is version JXOP01000000.
